# Lavage in Children

**Published:** 1888-03

**Authors:** 


LAVAGE IN CHILDREN.—Loey, in *Prog. Med.*, reports success in the employment of lavage in the case of nineteen infants from one to sixteen months old, troubled with dyspepsia. The stomach was first evacuated, then warm water, containing a small amount of salt, was introduced until it returned perfectly clear. Relief was frequently instantaneous. The operation repeated a few times daily or at intervals, suffices for recovery.

